# Does childhood psychological maltreatment encourage you to become a cyberbullying perpetrator? The mediating role of negative affect and the moderating role of meaning in life

**DOI:** 10.3389/fpsyg.2023.1223535

**Published:** 2023-09-28

**Authors:** Shujing Zhang, Yi Li, Min Cao, Yuxiao Liu, Zongkui Zhou

**Affiliations:** ^1^School of Psychology, Central China Normal University, Wuhan, China; ^2^Students’ Affairs Office, Shangqiu Normal University, Shangqiu, China; ^3^Henan Police College, Zhengzhou, China

**Keywords:** childhood psychological maltreatment, cyberbullying, meaning in life, negative affect, college students

## Abstract

**Objective:**

With the development of information and communication technology, cyberbullying among Chinese college students has become more frequent, bringing many negative consequences to both society and students themselves. Childhood psychological maltreatment may be one of the influencing factors of cyberbullying, but its internal mechanism remains poorly understood. This study aimed to explore the relationship between childhood psychological maltreatment and cyberbullying among college students and to further explore the mediating effect of negative emotion and the moderating effect of meaning in life.

**Methods:**

In this study, 656 college students (48.7% males) were recruited to complete anonymous questionnaires assessing their perceptions of child psychological maltreatment, negative affect, meaning in life and cyberbullying. SPSS23.0 and Hayes PROCESS macro for SPSS were used to conduct statistical analysis.

**Results:**

(1) Childhood psychological maltreatment was significantly positively associated with cyberbullying; (2) Negative affect played a partially mediating role between childhood psychological maltreatment and cyberbullying; and (3) Meaning in life moderated the direct association between childhood psychological maltreatment and cyberbullying and moderated the association between negative affect and cyberbullying.

**Conclusion:**

In this study, a moderated mediation model was constructed and the internal mechanism of childhood psychological maltreatment and cyberbullying among college students was found. The results provided both theoretical contributions and practical suggestions for preventing cyberbullying.

## 1. Introduction

The development of information and communication technologies (ICTs) has revolutionized the way in which people establish and maintain relationships and carry out daily communication. According to a report, there are 1.067 billion netizens in China, and the internet penetration rate had reached 75.6% by December 2022 (China Internet Network Information Center, 2023). Although ICTs provide many conveniences to people, they bring a series of social problems, such as increasing cyberbullying. Cyberbullying refers to “An aggressive, intentional act carried out by a group or individual, using electronic forms of contact, repeatedly and over time against a victim who cannot easily defend him or herself” (Smith et al., 2008), with the properties of spatial transcendence, temporal asynchrony, interpersonal anonymity, and self-disinhibition (Zhou and Liu, 2016). These properties make cyberbullying more likely to occur and minimize the punishment consequences for the cyberbullying perpetrator. Previous studies have shown that cyberbullying can lead to anxiety, depression, substance abuse, suicidal ideation, and other negative outcomes (Kowalski et al., 2014; Kwan et al., 2020). Given the increasing prevalence of cyberbullying among Chinese college students (Zhu et al., 2016; Leung et al., 2018), cyberbullying may become one of the most common forms of bullying among college students (Francisco et al., 2015). Therefore, it is essential and necessary to explore the mechanism of cyberbullying occurrence among college students.

Previous studies have shown that the influencing factors of cyberbullying mainly include individual factors and environmental factors (Ding et al., 2020). Individual factors include individual personality characteristics (Zhang et al., 2020), emotional state (Kowalski et al., 2014), self-related cognition, externalizing and internalizing problems, social competence, academic performance (Ding et al., 2020). Environmental factors mainly include parenting style (Hinduja and Patchin, 2022), family socioeconomic status (Yang et al., 2023), stressors (Islam et al., 2022), family environment, school climate, and peer status (Ding et al., 2020). It can be seen that family factors are important factors influencing individual cyberbullying (Guo et al., 2021; Li et al., 2022). Based on the view of the General Aggressive Model (GAM), aggressive behavior is more likely if one has received poor parenting or lived with coercive families (Allen et al., 2018). As a family factor, childhood psychological maltreatment refers to a series of inappropriate parenting practices adopted by caregivers in the course of children’s growth that hinder the normal development of children’s cognition, emotional and behavioral patterns, for instance, intimidation, belittlement, interference, indulgence, and neglect (Pan et al., 2010). It has a significant association with an individual’s aggressive behaviors (Miller et al., 2013; Qin et al., 2021; Zhang et al., 2021). Aggressive behavior is often manifested in the form of cyberbullying in college students. Therefore, we consider that childhood psychological maltreatment is positively related to cyberbullying among college students. Previous studies have shown that positive parenting practices discourage bullying, while those marked by rejection, chaos, and coercion are associated with increased bullying (Hinduja and Patchin, 2022), so it is valuable to explore the impact of childhood psychological abuse on cyberbullying among college students.

According to the GAM, situational factors are the environmental features that limit or promote cyberbullying (Kokkinos and Antoniadou, 2019). Situation factors can induce an individual’s aggressive behavior through the activation of hostile thoughts, negative affect, and hyperarousal (Vannucci et al., 2012). Individuals show more negative affect when they grow up if they usually suffered psychological maltreatment from their caregivers in their childhood (Gu et al., 2023). This is because negative life experience makes an individual more sensitive to negative information (Baugerud et al., 2016), and negative affect is a factor that can directly induce aggressive behavior (Allen et al., 2018). Therefore, childhood psychological maltreatment may induce negative affect and then likely give rise to cyberbullying.

However, not all individuals who have experienced childhood psychological maltreatment are involved in cyberbullying. Some protective factors may buffer the effects of childhood psychological maltreatment on cyberbullying. Meaning in life is a very important protective factor for individuals in adversity (Russo-Netzer et al., 2019), and it refers to the sense made of and significance felt regarding the nature of one’s being and existence (Steger et al., 2006). The experience of childhood psychological maltreatment is a kind of growth adversity for individuals, and the sense of life meaning can buffer its negative effects, such as reducing aggressive behavior (such as cyber bullying). Meaning in life can also buffer the negative impact of risk factors on individuals and play an important role in protecting the psychological adaptation of individuals (Kleiman et al., 2013). Therefore, as a stable protective factor, meaning in life is able to moderate the impact of risk factors on individual externalizing problems. Previous studies have proven that individuals with a higher level of meaning in life are less susceptible to negative experiences than those who feel meaningless (Czekierda et al., 2017). In contrast, low meaning in life is more likely to drive aggressive behavior to some extent (Tilburg et al., 2019). Thus, meaning in life buffers the effect of childhood psychological maltreatment on cyberbullying.

Moreover, according to the GAM, a person’s assessment of the situation may be affected by the person’s current internal state (Allen et al., 2018). When people have sufficient mental resources, they carefully reevaluate the event (i.e., consider alternative explanations), and aggression might not happen (Allen et al., 2018). Meaning in life is an important mental resource of people (Seon and Smith, 2023), and it is a very important protective factor for individuals in adversity (Russo-Netzer et al., 2019). That is, individuals with different levels of meaning in life could choose whether to commit aggressive behavior differently when faced with the same situation or emotional state. Higher meaning in life can help people deal with negative events or situations that may lead to aggressive behavior according to the dual-systems model of meaning in life (Wong, 2010, 2012). Similarly, higher meaning in life is better able to help people regulate negative affect and seldom cause cyberbullying (MacKenzie and Baumeister, 2014). Therefore, we conclude that meaning in life may moderate the relationship between childhood psychological maltreatment and cyberbullying, and it could moderate the relationship between negative affect and cyberbullying.

Previous studies have well illustrated the risk factors and protective factors of cyberbullying (Zhang et al., 2020; Islam et al., 2022), but few studies have discussed the mediating role of negative affect and the moderating role of meaning in life in the relationship between childhood psychological maltreatment and cyberbullying among college students. Therefore, we utilize a process-oriented approach to investigate the underlying mediating and moderating mechanism of childhood psychological maltreatment on cyberbullying. The study aims to answer how and under what conditions childhood psychological maltreatment affects cyberbullying. Based on the current theories and research conclusions, we propose the following hypotheses:

*Hypothesis 1*: Childhood psychological maltreatment is positively associated with cyberbullying among college students.

*Hypothesis 2*: Negative affect mediates the relationship between childhood psychological maltreatment and cyberbullying.

*Hypothesis 3*: Meaning in life moderates the association between childhood psychological maltreatment and cyberbullying and the association between negative affect and cyberbullying.

**Figure 1:**
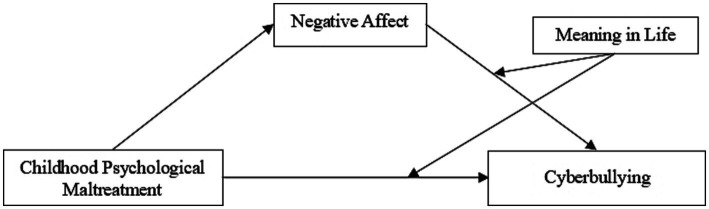
Conceptual model for the conditional process analysis.

A diagram of the conceptual model for the conditional process analysis is shown in Figure 1.

## 2. Materials and methods

### 2.1. Participants

Participants were recruited from 5 universities in Henan Province, China. A total of 720 Chinese undergraduate students participated in this study, and 64 participants were excluded because of extensive missing data, resulting in a final sample of 656 participants (48.7% males, 1 missing gender item), with 150 freshmen, 254 sophomores, 215 juniors, and 37 seniors.

### 2.2. Measures

#### 2.2.1. Cyberbullying inventory

Cyberbullying was assessed by the Cyberbullying subscale of the Cyberbullying and Victimization Inventory. This inventory was developed by Erdur-Baker and Kavsut (2007) and revised by Zhou et al. (2013) in the context of Chinese culture. The Cyberbullying subscale included 18 items, each of which was scored on a four-point ordinal scale (1 = never encountered, 2 = 1–2 times, 3 = 3–5 times, and 4 = more than 5 times). A sample item is “I have spread rumors about someone on the Internet.” Participants were instructed to rate how often they had experienced the described events during the past semester. Higher average scores calculated by all items represent a higher frequency of cyberbullying. Previous studies have demonstrated the scale’s good reliability and validity for Chinese college students (Zhou et al., 2013). In this study, the measure demonstrated good reliability (α = 0.95).

#### 2.2.2. Childhood psychological maltreatment scale

The Child Psychological Maltreatment Scale developed by Pan et al. (2010) was utilized to measure childhood psychological maltreatment in this study. It includes 23 items and consists of five dimensions: insults, threats, interference, neglect and indulgence. A sample item is “My parents abused me when I did not expect it.” Participants were required to recall their real interactions with caregivers before the age of 18 to determine the degree of psychological maltreatment they suffered through a five-point scale (0 = none, 1 = very few, 2 = sometimes, 3 = often, and 4 = always). A higher average score indicated more psychological maltreatment events experienced by the participants. In this study, the measure demonstrated good reliability (α = 0.92).

#### 2.2.3. Negative affect scale

Negative Affect was assessed by the Negative Affect Subscale of the Positive and Negative Affect Scale. This inventory was developed by Watson et al. (1988) and revised by Qiu et al. (2008) in the context of Chinese culture. It contains nine words that describe negative affect experiences in daily life, such as “irritable,” and participants were required to choose the number that most accurately matched the intensity of their effect on a five-point scale (1 = none or very mild, 2 = a little, 3 = moderate, 4 = strong, and 5 = very strong). Higher average scores indicated more experiences of negative affect. In the present study, the measure had adequate internal consistency (α = 0.86).

#### 2.2.4. Meaning in life questionnaire

Meaning in life was measured by the Chinese version of the Meaning in Life Questionnaire (Steger et al., 2006), which was revised by Liu and Gan (2010). The scale includes 9 items assessing two dimensions of meaning in life: the presence of meaning (e.g., “I know the meaning in my life”) and search for meaning (e.g., “I’m looking for meaning in my life”). Participants indicated their meaning in life on a seven-point scale from 1 (strongly disagree) to 7 (strongly agree). Higher average scores indicate greater perception of personal goals and values and a stronger tendency to pursue the value of life. In the present study, the measure had adequate internal consistency (α = 0.82).

#### 2.2.5. Control variables

Previous studies found that there were significant gender differences in childhood psychological maltreatment, cyberbullying and meaning in life (Zhou et al., 2013; David et al., 2018; Russo-Netzer et al., 2019). Gender differences in childhood psychological maltreatment (*t* = 4.78, *p* < 0.05), negative affect (*t* = 6.02, *p* < 0.05), cyberbullying (*t* = 6.52, *p* < 0.05) and meaning in life (*t* = −3.00, *p* < 0.05) were found in this study, and there are also significant gender differences in cyberbullying(*F* = 3.46, *p* = 0.016): juniors (*M* = 1.17, *SD* = 0.43) had significantly higher scores than sophomores (*M* = 1.09, *SD* = 0.29, *p* = 0.004) and seniors (*M* = 1.05, *SD* = 0.09, *p* = 0.031). Therefore, gender and grade were controlled in our statistical analysis.

### 2.3. Procedure

**Figure 2:**
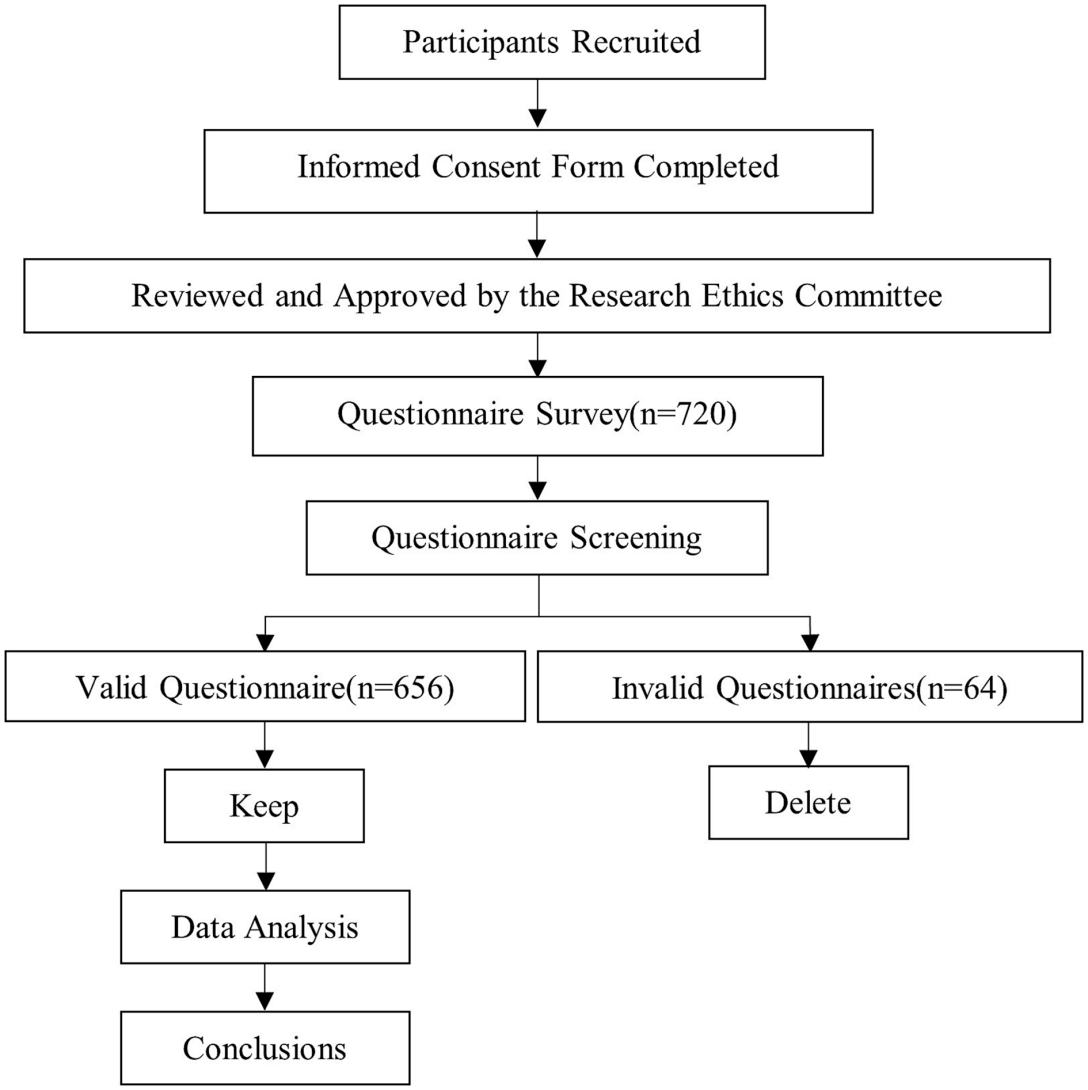
General diagram of the study process.

All participants completed an informed consent form prior to completing the questionnaire, and this study was reviewed and approved by the Research Ethics Committee of the corresponding author’s institution. Participants completed a set of questionnaires during regular school time on demographic variables, cyberbullying, childhood psychological maltreatment, negative affect, and meaning in life. After finishing the survey, each participant received a small gift (a mobile phone holder) as a reward. We placed the Childhood Psychological Maltreatment Scale at the end of the questionnaire to avoid the priming effect of recalling adverse childhood experiences on other variables. General diagram of process in this study is shown in Figure 2.

### 2.4. Statistical analyses

In this study, all analyses were conducted in SPSS 23.0 and the SPSS macro program PROCESS. First, we used Harman’s single-factor test (Podsakoff and Organ, 1986) to check for common-method bias for the use of self-report questionnaires. Second, we conducted descriptive statistics and correlation analysis to investigate the bivariate associations among variables. Third, the SPSS macro PROCESS (Model 4) was conducted to test the mediation model, and we examined the mediating role of negative affect in the mediation model (i.e., from childhood psychological maltreatment to cyberbullying). Finally, the SPSS macro PROCESS (Model 15) was conducted for conditional process analysis to confirm whether meaning in life moderated the mediation model.

## 3. Results

### 3.1. Check for common method bias

The measures of variables in this study were based on college students’ self-reports. Thus, we utilized Harman’s one-factor test (Podsakoff and Organ, 1986) to determine the extent to which correlations among the variables in this study may have resulted from common method variance. Unrotated factor analysis indicated that there were 9 common factors being extracted of the characteristic value is greater than 1, and the first common factor explained 26.7% of the total variance, it was less than 40% (Podsakoff et al., 2003). The results implied that common method bias in the present study is unlikely to be a serious problem.

### 3.2. Preliminary analysis

With reference to the conservative criteria for cyberbullying used in a previous study (Brochado et al., 2017), scores higher than the average score indicated that participants had perpetrated cyberbullying. The prevalence of cyberbullying in this study is 38.4%. Childhood psychological maltreatment with an average score of ≥1 indicated that participants had experienced psychological maltreatment in childhood (Jin et al., 2017), and the prevalence of childhood psychological maltreatment in this study was 32.5%. The means, standard deviations, and correlation coefficients are shown in Table 1: childhood psychological maltreatment was significantly positively associated with negative affect and cyberbullying, and meaning in life was significantly negatively correlated with childhood psychological maltreatment, negative affect, and cyberbullying. Therefore, Hypothesis 1 was supported.

**Table 1:** Descriptive statistics and correlations among all study variables.

	*M*	*SD*	1	2	3	4
1. CPM	0.82	0.57	1			
2. NA	1.82	0.63	0.36^***^	1		
3. ML	4.86	0.98	−0.12^**^	−0.22^***^	1	
4. CB	1.12	0.33	0.43^***^	0.36^***^	−0.20^***^	1

*N* = 656. ^**^p < 0.01, ^***^p < 0.001. CPM, childhood psychological maltreatment, NA, negative affect; ML, meaning in life; CB, cyberbullying, the same below.

### 3.3. Testing for the mediating role of negative affect

**Figure 3:**
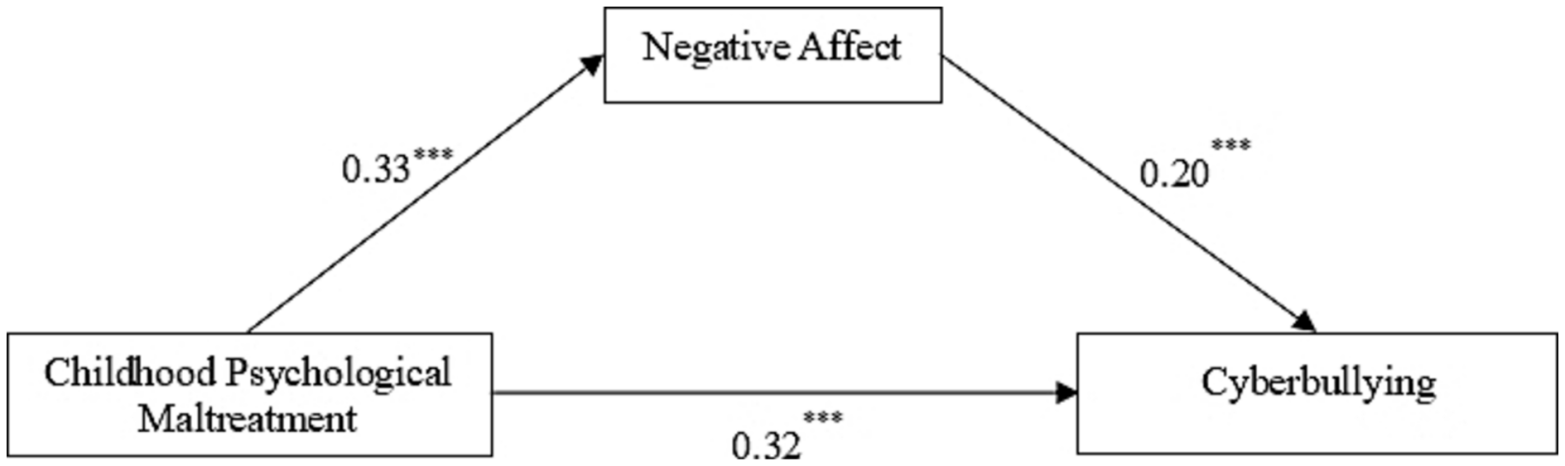
The mediating role of negative affect. The path coefficients were reported.

After controlling gender and grade, we used the PROCESS macro (Model 4) to examine the mediating role of negative affect in the relationship between childhood psychological maltreatment and cyberbullying. The results (see Table 2) showed that after controlling for gender and grade, childhood psychological maltreatment was positively associated with cyberbullying (β = 0.39, *p* < 0.001) and negative affect (β = 0.33, *p* < 0.001), and negative affect was positively associated with cyberbullying (β = 0.20, *p* < 0.001). Moreover, when both childhood psychological maltreatment and negative affect predicted cyberbullying, negative affect had a significant positive predictive effect on cyberbullying (β = 0.20, *p* < 0.001), and the positive predictive effect of childhood psychological maltreatment on cyberbullying was still significant (β = 0.32, *p* < 0.001). After controlling for students’ grades and gender, childhood psychological maltreatment positively predicted negative affect, which in turn predicted cyberbullying (Figure 3). Negative affect plays a partially mediating role between childhood psychological maltreatment and cyberbullying (indirect effect = 0.07, SE = 0.02, 95% confidence interval = 0.04 to 0.10). The mediation effect accounted for 16.82% of the total effect of childhood psychological maltreatment on cyberbullying. Therefore, Hypothesis 2 was supported.

**Table 2:** Testing the mediation effect of negative affect.

Criterion	Predictors	*R*	*R^2^*	*F(df)*	*β*	*t*
CB	Gender	0.47	0.22	59.96 (3)	−0.38	−5.31^***^
	Grade				0.07	1.72
	CPM				0.39	11.12^***^
NA	Gender	0.40	0.16	41.58 (3)	−0.37	−4.94^***^
	Grade				0.08	1.92
	CPM				0.33	8.90^***^
CB	Gender	0.50	0.25	54.48 (4)	−0.30	−4.29^***^
	Grade				0.05	1.34
	CPM				0.32	8.92^***^
	NA				0.20	5.48^***^

### 3.4. Testing for conditional process modeling

We conducted Model 15 of the SPSS PROCESS macro program to examine the moderated mediation model. Childhood psychological maltreatment significantly and positively predicted negative affect (β = 0.33, *p* < 0.001) after controlling for gender and grade (Table 3). In addition, the interaction of childhood psychological maltreatment and meaning in life had a significant effect on cyberbullying (β = −0.17, *t* = −5.06, *p* < 0.01), and the interaction of negative affect and meaning in life also had a significant effect on cyberbullying (β = −0.12, *t* = −3.71, *p* < 0.01) (Table 3). These findings indicated that both the relationship between childhood psychological maltreatment and cyberbullying and the relationship between negative affect and cyberbullying were moderated by meaning in life.

**Table 3:** Moderated mediation model testing.

Regression equation (N = 655)		Fitting index			Coefficient significance	
Outcome variables	Predictive variable	*R*	*R* ^2^	*F(df)*	*β*	*t*
NA		0.40	0.16	41.58 (3)		
	Gender				−0.37	−4.94^***^
	Grade				0.08	1.92
	CPM				0.33	8.90^***^
CB		0.56	0.31	42.46 (7)		
	Gender				−0.27	−3.92^***^
	Grade				0.06	1.57
	CPM				0.31	8.88^***^
	NA				0.16	4.42^***^
	ML				−0.10	−2.91^***^
	CPM × ML				−0.17	−5.06^***^
	NA × ML				−0.12	−3.71^***^

**Figure 4:**
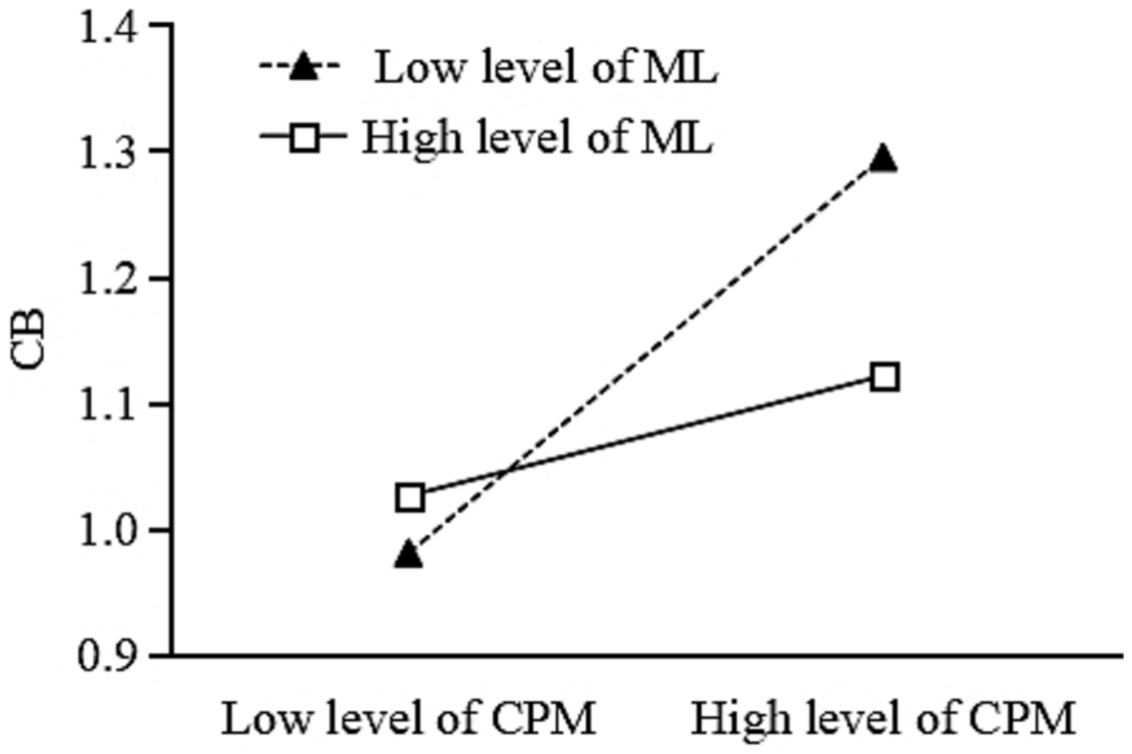
The moderating effect of meaning in life on the relationship between childhood psychological maltreatment and cyberbullying.

**Figure 5:**
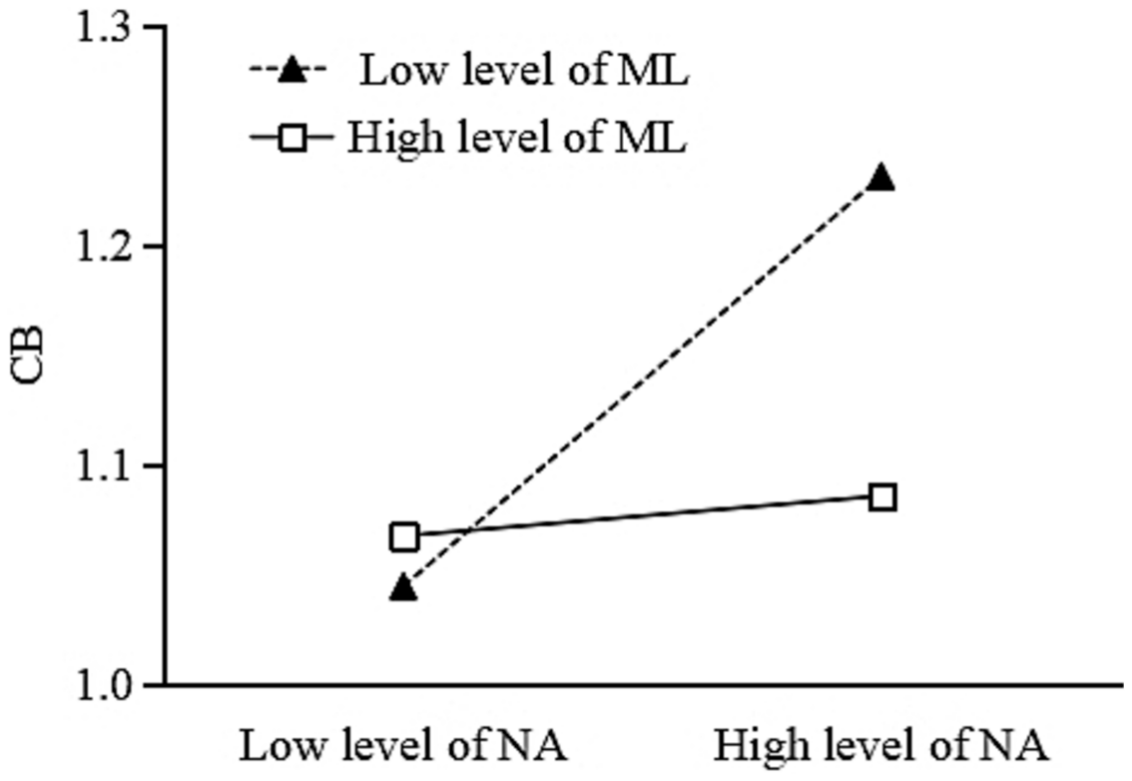
The moderating effect of meaning in life on the relationship between negative affect and cyberbullying.

To better understand the moderating effect of meaning in life, a simple slope analysis was conducted (see Figures 4, 5). As shown in Figure 4, for participants with low meaning in life (*M - 1SD*), childhood psychological maltreatment had a significant positive predictive effect on cyberbullying (simple slope = 0.48, *t* = 9.91, *p* < 0.001), while for participants with high meaning in life (*M + 1SD*), childhood psychological maltreatment also had a positive predictive effect on cyberbullying, but the effect was relatively smaller (simple slope = 0.14, *t* = 3.00, *p* = 0.003). The results showed that meaning in life appeared to play a protective role between childhood psychological maltreatment and cyberbullying (Table 4).

**Table 4:** Direct effects and mediating effects on different levels of meaning in life.

	ML	Effect value	SE	Low 95% CI	Upper 95% CI
Direct effects	3.88 (*M*–*1SD*)	0.48	0.05	0.38	0.57
	4.86 (*M*)	0.31	0.03	0.24	0.38
	5.84 (*M + 1SD*)	0.14	0.05	0.05	0.24
Mediating effects of NA	3.88 (*M*–*1SD*)	0.09	0.02	0.05	0.14
	4.86 (*M*)	0.05	0.01	0.03	0.08
	5.84 (*M* + 1*SD*)	0.01	0.02	−0.02	0.04

As shown in Figure 5, for participants with low meaning in life (1 SD below the mean), negative affect has a significant positive predictive effect on cyberbullying (β = 0.28, *t* = 5.75, *p* < 0.001). Nevertheless, for participants with high meaning in life (1 SD above the mean), negative affect does not have a positive predictive effect on cyberbullying (β = 0.04, *t* = 0.72, *p* = 0.47). The results showed that meaning in life appeared to play a protective role between childhood psychological maltreatment and cyberbullying. That is, a high level of meaning in life appeared to play a protective role in college students’ healthy online behavior. In summary, the indirect effect of childhood psychological maltreatment on cyberbullying through negative affect was observed when meaning in life was low but not when meaning in life was high (Table 4). These results provided support for Hypothesis 3.

## 4. Discussion

The General Aggression Model maintains that an individual’s aggressive behaviors are the result of the interaction between person factors and situation factors (Anderson and Bushman, 2002). According to this model, college students’ aggressive behaviors on the internet (in our study, cyberbullying) are likely associated with both personal factors (in our study, negative affect and meaning in life) and situational factors (in our study, childhood psychological maltreatment). Therefore, in this study, we conducted a moderated mediation model (analysis of negative affect as a mediator and meaning in life as a moderator) to reveal how and when childhood psychological maltreatment predicted cyberbullying.

The results showed that childhood psychological maltreatment could not only directly predict cyberbullying in college students but also indirectly affect cyberbullying through the mediating effect of negative affect. In addition, meaning in life can moderate the direct path and the mediating effect of negative affect. Specifically, for participants with lower meaning in life, childhood psychological maltreatment has a stronger direct and indirect effect on cyberbullying. However, for participants with higher meaning in life, negative affect no longer has a significant indirect effect on cyberbullying. The results improve the understanding of the influence of childhood psychological maltreatment on cyberbullying, as well as the specific internal mechanisms.

### 4.1. The relationship between childhood psychological maltreatment and cyberbullying

Childhood psychological maltreatment was found to have a positive effect on cyberbullying among college students, which is consistent with previous research on adolescents (Chen et al., 2020; Jin et al., 2020; Sun et al., 2020). College students who have experienced childhood psychological maltreatment have accumulated a large number of aggression-related clues (such as provocation and frustration, etc.) through long-term interaction with their parents. When individuals encounter certain clues, they automatically conduct an immediate assessment of the situation. This assessment process is spontaneous and unconscious and requires almost no cognitive effort. Individuals can directly and automatically initiate attacks based on the original cognitive script when they think they are in a threatening environment (Anderson and Bushman, 2002; Allen et al., 2018). Therefore, this study confirms previous research and concludes that childhood psychological maltreatment is able to positively predict cyberbullying in college students and provides empirical support for the GAM.

### 4.2. The mediating effect of negative affect

This study found that negative affect could mediate the association between childhood psychological maltreatment and cyberbullying, supporting Hypothesis 2. Individuals who have experienced childhood psychological maltreatment are prone to form latent negative self-schemas (Bai et al., 2021). Negative experiences activate negative metacognitive processes that make individuals more sensitive to negative events, resulting in a negative affect state for a long time (Østefjells et al., 2017). Moreover, the anonymity and convenience of the network environment provide a platform for alleviating negative affect. The virtual world enables individuals to hide their true identities, thereby reducing the possibility of being negatively evaluated or attacked by others. That is, the activation level of the behavioral inhibition system in online social interaction is lower than that in real social interaction (Yen et al., 2012), which may prompt individuals to engage in inappropriate behaviors online (such as cyberbullying). Therefore, college students who have experienced childhood psychological maltreatment are more likely to alleviate their accumulated negative affect in real interpersonal conflicts by perpetrating cyberbullying. In other words, the higher the level of childhood psychological maltreatment, the more likely individuals are to induce negative affect and thus more likely to perpetrate cyberbullying. This result provides a clear path for how to carry out intervention work to prevent cyberbullying in college students.

### 4.3. The moderating effect of meaning in life

The present study documents that high meaning in life could weaken the direct effect of childhood psychological maltreatment on cyberbullying, and the indirect effect through negative affect, making Hypotheses 2 and 3 valid. These findings provide evidence for the dual-systems model (Wong, 2010), which suggests that meaning in life helps individuals in adversity overcome difficulties to protect them from potential threats (Wong, 2012). Despite experiencing childhood psychological maltreatment, college students with high meaning in life can deal with negative events or situations and effectively reduce potential cyberbullying activities. One of the core functions of meaning in life is to stimulate one’s self-control ability, regulate emotions, and further guide behavior (MacKenzie and Baumeister, 2014). Individuals with high meaning in life are better able to exercise self-control and regulate their emotions and behavior than those with low meaning in life (Lengua et al., 2008); thus, they are unlikely to perpetrate cyberbullying. However, individuals with low meaning in life have lower self-control and are more likely to be driven by internal impulses and instincts (Zhang et al., 2019), making it easier for them to perpetrate cyberbullying. Thus, college students with high meaning in life could handle the setbacks and the negative affect from childhood psychological maltreatment experience.

### 4.4. Strengths, limitations, and future directions

This study has the following advantages. First, a moderated mediation model is constructed in this study to reveal the impact and mechanism of childhood psychological maltreatment, an important family factor, on cyberbullying. The results not only provide empirical support for the General Aggression Model but also provide practical suggestions for preventing cyberbullying among college students. According to our findings, combating cyberbullying requires systematic and conjugate interventions involving families, schools and students themselves. Specifically, parents should avoid psychological maltreatment of their children in their childhood from the source, and adopt reasonable parenting methods, which are conducive to their children’s lifelong development. Second, schools can incorporate cyberbullying prevention into anti-bullying plans, including schoolwide anti-bullying policies, promotion and education activities to prevent cyberbullying and curriculum-based activities. The network center in the university could carry out network counseling lectures so that students can use the network safely and realize the risk factors that they possibly face in the network space. The student affairs office can closely pay attention to emotional status (such as anger, anxiety, panic, etc.) of students who have experienced psychological maltreatment in childhood in combination with an annual psychological survey and provide effective emotional counseling and behavioral guidance to prevent students from carrying out cyberbullying. Moreover, this study also showed that meaning in life, as an important protective factor, can moderate the impact of negative factors on cyberbullying. This result can provide some enlightenment for the prevention and intervention of cyberbullying among college students, which is to reduce the occurrence of cyberbullying by intervening meaning in life of college students. For example, mindfulness and expressive art therapy can be used to interfere with the meaning of an individual’s life to reduce or eliminate cyberbullying (Manco and Hamby, 2021). In addition, universities should clearly recognize the importance of life education and enhance students’ meaning in life, which could weaken the negative effects of childhood psychological maltreatment and ultimately reduce latent cyberbullying.

This study also has some limitations as follows. First, the use of cross-sectional studies has limitations in explaining the inherent causal relationship between variables. In future studies, longitudinal studies or randomized controlled experiments can be used to explore the impact mechanisms of childhood psychological maltreatment on cyberbullying. Second, methodological bias is inevitable in survey data. There may be subjective bias in this study since the data are collected from self-report questionnaires that request that participants recall their childhood experiences. In future studies, a combination of peer ratings and self-ratings could be utilized to test the impact mechanisms of childhood psychological maltreatment on online bullying. Third, this study investigated the degree of psychological maltreatment experienced by college students in childhood but did not explore their current status of psychological maltreatment. Future studies can compare the degree of past and present experiences of psychological maltreatment among participants and explore the mechanisms of change. Forth, due to the reasons such as the need for internship and job hunting, there were only a small number of senior students on campus. Other senior students who are not in school have no suitable time and occasion to complete a rigorous questionnaire survey. As a result, there were only 37 valid questionnaires for senior students in this study. This is the deficiency of this study, and the future research will pay attention to this problem.

## 5. Conclusion

The following conclusions can be drawn from this study: (1) childhood psychological maltreatment was significantly positively associated with cyberbullying among Chinese college students; (2) negative affect plays a partially mediating role between childhood psychological maltreatment and cyberbullying; and (3) meaning in life moderates the direct association between childhood psychological maltreatment and cyberbullying and the association between negative affect and cyberbullying.

## Data availability statement

The raw data supporting the conclusions of this article will be made available by the authors, without undue reservation.

## Author contributions

SZ and YiL conceived the study and collected the data. MC provided valuable suggestions for the revision of the paper. SZ and YuL analyzed and processed the data. ZZ provided critical guidance for the concept and design of the study. All authors contributed to the article and approved the submitted version.

## Funding

This study was financially supported by the Research Program Funds of the Collaborative Innovation Center of Assessment toward Basic Education Quality at Beijing Normal University in China (no. 2021-04-003-BZPK01) and the Annual Program of Philosophy and Social Sciences of Henan Province (no. 2022BJY026).

## Conflict of interest

The authors declare that the research was conducted in the absence of any commercial or financial relationships that could be construed as a potential conflict of interest.

The handling editor XC declared a past collaboration with the author ZZ.

## Publisher’s note

All claims expressed in this article are solely those of the authors and do not necessarily represent those of their affiliated organizations, or those of the publisher, the editors and the reviewers. Any product that may be evaluated in this article, or claim that may be made by its manufacturer, is not guaranteed or endorsed by the publisher.
